# Telescoping Intestine in an Adult

**DOI:** 10.1155/2013/292961

**Published:** 2013-07-24

**Authors:** Khaldoon Shaheen, Naseem Eisa, Abdul Hamid Alraiyes, M. Chadi Alraies, Srinivas Merugu

**Affiliations:** ^1^Department of Hospital Medicine, Institute of Medicine, Cleveland Clinic, Cleveland, OH 44195, USA; ^2^Department of Pulmonary, Critical Care and Environmental Medicine, Tulane University Health Sciences Center, New Orleans, LA 70118, USA; ^3^Department of Medicine, Case Western Reserve University St. Vincent Charity Medical Center, Cleveland, OH 44115, USA

## Abstract

Protrusion of a bowel segment into another (intussusception) produces severe abdominal pain and culminates in intestinal obstruction. In adults, intestinal obstruction due to intussusception is relatively rare phenomenon, as it accounts for minority of intestinal obstructions in this population demographic. Organic lesion is usually identifiable as the cause of adult intussusceptions, neoplasms account for the majority. Therefore, surgical resection without reduction is almost always necessary and is advocated as the best treatment of adult intussusception. Here, we describe a rare case of a 44-year-old male with a diffuse large B-cell lymphoma involving the terminal ileum, which had caused ileocolic intussusception and subsequently developed intestinal obstruction requiring surgical intervention. This case emphasizes the importance of recognizing intussusception as the initial presentation for bowel malignancy.

## 1. Introduction

Protrusion of a bowel segment into another produces severe abdominal pain and culminates in intestinal obstruction. In adults, intestinal obstruction due to intussusception is relatively rare phenomenon, as it accounts for minority of intestinal obstructions in this population demographics. In this case, we provide diagnostic, therapeutic, and prognostic keys to bowel intussusception in adults.

## 2. The Case

**Figure 1:**
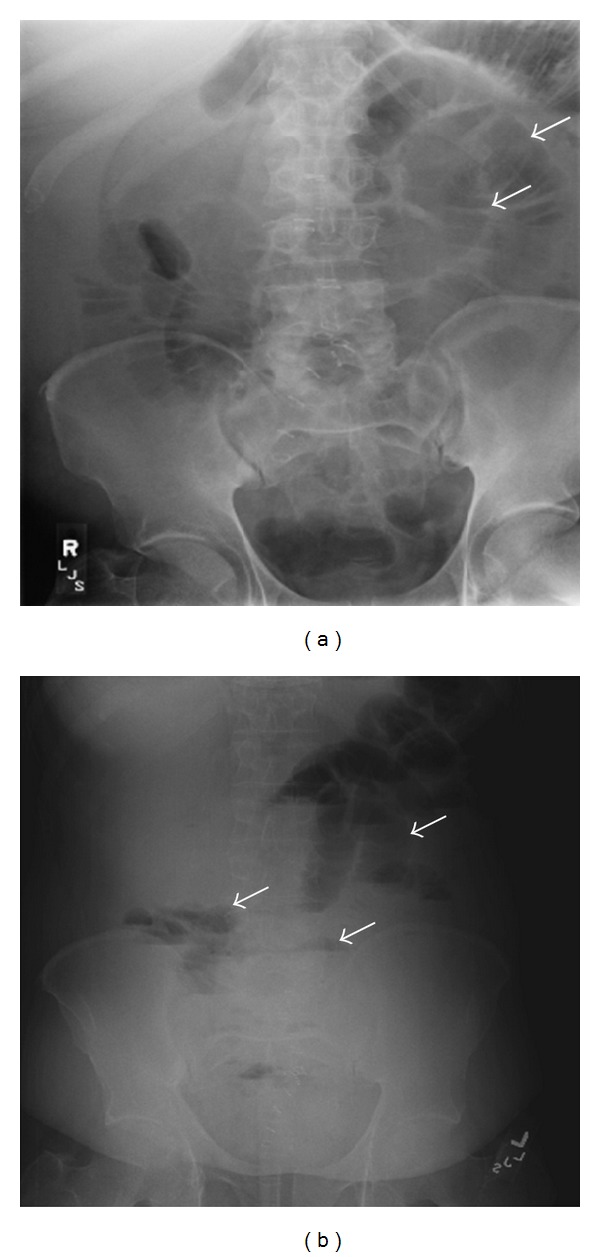
X-ray abdomen. (a) Supine abdominal radiograph shows dilated small bowel loops and absence or paucity of gas in the large bowel due to distal small bowel obstruction. (b) Upright abdominal radiograph shows multiple air-fluid levels in upper and central abdomen that represent dilated proximal and central fluid-filled small bowel due to distal small bowel obstruction (arrows).

**Figure 2:**
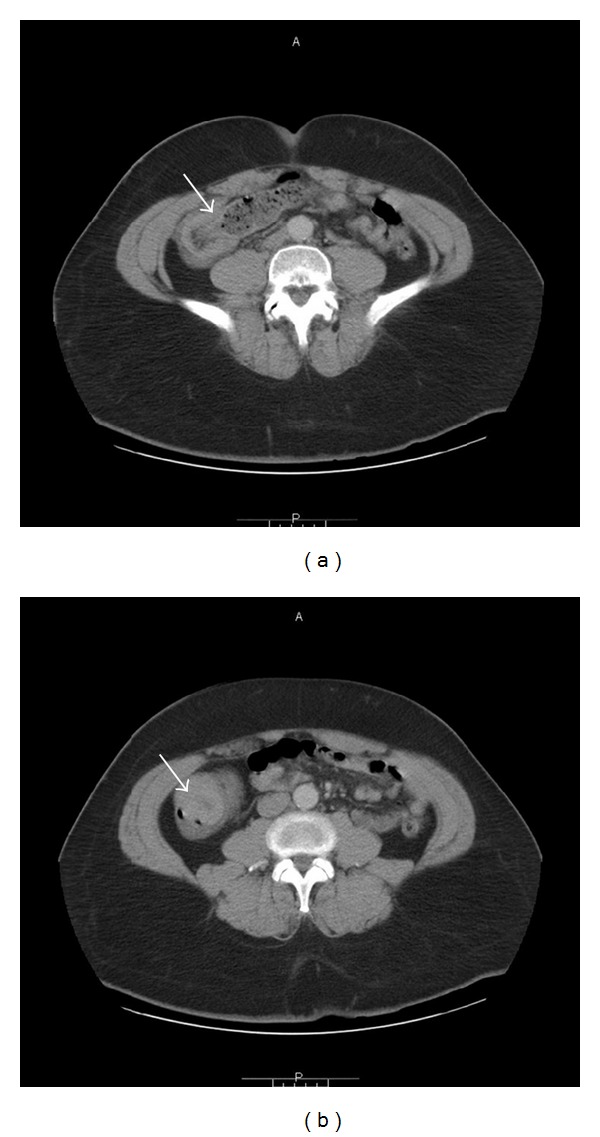
A computed tomography (CT) scan of the abdomen. (a) Axial CT scan at level of iliac crests shows a longitudinal sectional image of the intussusception. A portion of the terminal ileum is inside the cecum and far from the invaginated ileal mesentery that separates the walls of the two bowel segments (arrow). (b) Cross-sectional image of the midportion of intussusception (arrow) illustrates small bowel invagination through the ascending colon just above the cecum (target sign). These findings are consistent with ileocolic intussusception.

**Figure 3:**
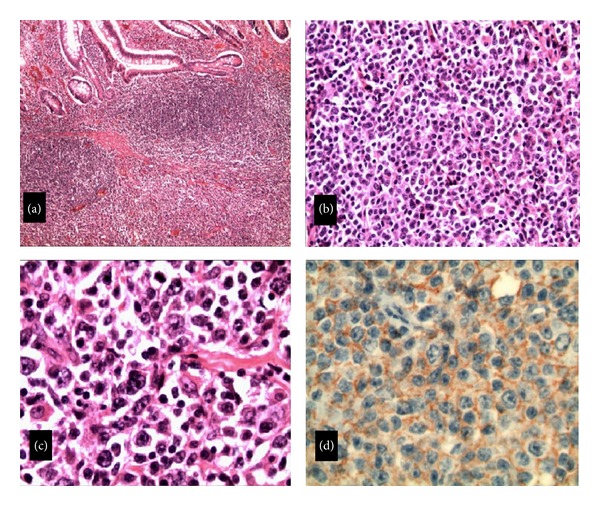
Diffuse large B-cell lymphoma (DLBCL): the tumor composed of diffuse infiltration of large lymphoid cells in all wall layers of the affected terminal ileum. (a) Staining with hematoxylin-eosin, magnification ×40. (b) Staining with hematoxylin-eosin, magnification ×200. (c) Staining with hematoxylin-eosin, magnification ×400. (d) Immunoperoxidase staining for CD20, magnification ×400.

A 44-year-old construction worker man, with no past medical problems, was admitted with two-month history of recurrent and progressively worsening abdominal pain and bleeding per rectum. Pain was colicky in nature and well localized to lower abdomen causing significant distress and insomnia. He denied similar symptoms in the past and he never had a colonoscopy. His condition-associated with loss of weight of about 20 pounds. Patient denied hematuria, frequency, and dysuria. There was no history of fever. He has no medications and denied using NSAIDs. His social history was significant for smoking (20 pack-year-histories) and occasional alcohol use. He denied any illicit drug abuse. His temperature was 36.4°C, blood pressure 103/63 mmHg, heart rate 96/minute, respiratory rate 18/minute, and SaO_2_ of 97% on room air. He was in mild distress secondary to abdominal pain. Abdominal examination was significant for mild tenderness in the suprapubic region and left iliac fossa with no rebound tenderness. Rectal exam was positive for fecal occult blood. Remainder of the physical exam was unremarkable. Laboratory investigation was significant for WBC 13.400 cells/mm^3^ with normal differential, hemoglobin 7.4 g/dL, hematocrit 23.1%, and platelet count 701,000 cells/mm^3^. Abdominal X-ray (Figure 1) showed dilated small bowel loops and multiple air-fluid levels suggesting distal small bowel obstruction. This was followed with computed tomography (CT) of the abdomen which revealed ileocolic intussusception (Figure 2). Surgery was consulted and patient was taken to operating room where a right hemicolectomy and extended ileac resection was performed. Surgery revealed a thickened mass involving the terminal ileum responsible for the ileocolic intussusception. Histopathology of the mass showed a diffuse large B-cell lymphoma involving the terminal ileum (Figure 3). No systemic lymphadenopathy was found suggesting a primary gastrointestinal non-Hodgkin's lymphoma. HIV test was negative. His postoperative course was uneventful, and later he was discharged home in stable condition.

## 3. Discussion

Intussusception is an invagination or “telescoping” of a proximal segment of bowel into the lumen of a distal segment leading to obstruction and compromise of mesenteric blood flow, with resultant ischemia of the bowel wall [1]. Any intraluminal lesion (leading point) is able to trigger an intraluminal invagination finally causing an intussusception. Subsequent peristaltic bowel activity produces an area of sequence constriction and relaxation, thus telescoping the leading point through the distal bowel lumen. 

Intussusception is commonly seen in children and reported as the second most common abdominal emergency in children, trailing only appendicitis. Adult intussusception, however, accounts only for 5% of all cases of intussusception and 1% of all cases of bowel obstruction in adults [2]. The mean age for intussusception is 50 years of age. Incidence is about the same in males and females [2]. 

Intussusceptions can be categorized into four types: enteroenteric, colocolic, enterocolic, and ileocecal. Enterocolic intussusception is the most common type [2]. In contrast to the pediatric population, where intussusception is usually idiopathic or secondary to viral illness, an organic lesion (leading point) is usually the identifiable culprit in adults. The vast majority (90%) of cases is due to neoplasm and the remainders (10%) are idiopathic. Tumors are 80% benign: that is, adhesions, lymphoid hyperplasia, trauma, lipomas, leiomyomas, Meckel's diverticulum, gastrointestinal stromal tumors, hemangiomas, or Peutz-Jegher adenoma. Only few cases (less than 20%) of small intestinal intussusceptions are malignant. In contrast, the colon is more likely (60%) to have malignant lesion as the cause of intussusception; two-thirds are due to primary colon adenocarcinoma and one-third is due to malignant lymphoma—the two constitute the most common malignant lesions in the colon [3]. 

An increased incidence of intussusception has been reported in patients with acquired immune deficiency syndrome (AIDS). This is due to the high incidence of infectious and neoplastic conditions like lymphoid hyperplasia, Kaposi's sarcoma, and non-Hodgkin's lymphoma. In HIV patients, lymphoma accounts for about 10% of all malignancies. It is thus not surprising that this population, more so than other population groups, is more frequently referred to surgery for abdominal complaints [4]. In our case, a 53-year-old male with diffuse large B-cell lymphoma involving the terminal ileum and had caused an ileocolic intussusception. Our literature search revealed that few cases of B-cell lymphoma, presenting as intussusceptions of terminal ileum, in HIV-seronegative adults, have ever been reported [5–8].

Intussusception in adults often present as chronic, intermittent abdominal pain associated with intermittent partial bowel obstruction which can cause nausea, vomiting, melena, weight loss, fever, and constipation [9]. Abdominal masses are palpable in 24%–42% of patients, and identification of a shifting mass or one that is palpable only when symptoms are present is suggestive of intussusception [3]. The relatively low incidence and varied presentation, both, account for the difficulty in making diagnosis of intussusception before surgery. Reijnen et al. reported a preoperative diagnostic rate of 50% [10], while Eisen et al. reported a lower rate of 40.7% [11].

A number of different radiologic methods have been described as useful in the diagnosis of intussusception. Plain abdominal films are typically the first diagnostic tool [12]; such films usually demonstrate signs of intestinal obstruction and suggest location of obstruction. Upper gastrointestinal series may show a “stacked coins” or “coiled spring” appearance [11]. Barium enema examination may be useful in patients with colonic or ileocolic intussusception in which a “cup-shaped” filling defect is a characteristic finding [11]. Ultrasonography is another useful measurement in the diagnosis of intussusceptions. The characteristic sonographic findings are the “target” or “doughnut” signs on the transverse view and the “pseudo-kidney” sign or “hay-fork” sign in the longitudinal view [13]. Certainly, an experienced radiologist is required to confirm such findings. Abdominal CT has been reported to be the most useful tool for diagnosis of intestinal intussusception and is superior to other contrast studies, ultrasonography or endoscopy [14]. A “target sign” may be seen on CT on perpendicular view (Figure 2(b)), while the intussusception will appear as a sausage-shaped mass when the CT beam is parallel to the longitudinal axis (Figure 2(a)). The distended loop of bowel (intussuscipiens) has a thickened wall because it represents two layers of bowel. 

While pediatric intussusception is usually due to a benign etiology and can usually be managed with nonoperative reduction (use of barium or air-contrast enemas), surgical resection without reduction is almost always necessary and is advocated as the best treatment of adult intussusception, given the high percentage of associated malignancy [14]. Nevertheless, some authors have recommended a selective approach to resection, particularly for small bowel intussusceptions, as the lower malignancy rate for small bowel intussusception makes the argument for initial resection less convincing. Furthermore, the choice of using a laparoscopic or open approach for small bowel resection depends on the clinical condition of the patient, the location and extent of intussusception, the possibility of underlying disease, and the availability of experienced surgeons [15].

The overall five-year survival for a primary gastrointestinal lymphoma is 38%. Curative resections yielded a survival of 60% regardless of site while palliative resections offered only a 17% chance of cure. As expected, survival was inversely proportional to extent of nodal spread. Postoperative radiotherapy is recommended for residual disease [16].

## 4. Conclusion

Intussusception in adults is a rare entity and diagnosis may be challenging because of nonspecific symptoms. Clinicians should be familiar with the various treatment options, because the real cause of the intussusception often is accurately diagnosed by laparotomy. CT is the most useful imaging modality in the diagnosis of intussusception. Treatment usually requires resection of the involved bowel segment. Reduction can be attempted in small-bowel intussusception if the segment involved is viable or malignancy is not suspected; however, a more careful approach is recommended in colonic intussusception because of a significantly higher chance of malignancy.
